# Adapting DeepLabV3+ for biopsy cervical cancer lesion segmentation

**DOI:** 10.3389/fdgth.2026.1776155

**Published:** 2026-05-11

**Authors:** Rose Nakasi, Cosmas Wamozo, Solomon Nsumba, Benjamin Rukundo, Tonny Okecha, Byron Mubiru, Chodrine Mutebi

**Affiliations:** 1 Artificial Intelligence Health Lab, Department of Computer Science, Makerere University, Kampala, Uganda; 2 Department of Computer Science, Makerere University, Kampala, Uganda; 3 Department of Pathology, Uganda Cancer Institute, Kampala, Uganda

**Keywords:** cervical cancer, deep learning, DeepLabV3+, digital pathology, histopathology, resource constrained settings, semantic segmentation, smartphone microscopy

## Abstract

**Introduction:**

Cervical cancer remains a leading cause of cancer mortality in resource-constrained settings, where access to advanced digital pathology equipment is severely limited. Automated histopathological image segmentation offers a potential pathway to improve diagnostic access, but practical solutions combining affordable hardware with robust deep learning remain underdeveloped.

**Methods:**

We present an approach combining smartphone-assisted microscopy with DeepLabV3+ architecture for precise segmentation of cervical cancer lesions in H&E-stained histopathological images. A custom smartphone adapter and the Ocular data collection app were used for standardized image acquisition. A DeepLabV3+ model with ResNet34 encoder was developed and validated on 5,966 histopathological images collected from the Uganda Cancer Institute, targeting 21 distinct histopathological feature classes using a combined BCE and Dice loss function with memory-efficient training on an NVIDIA RTX 3090.

**Results:**

On a held-out validation set drawn from the same institutional dataset, the system achieves a mean Intersection over Union (IoU) of 75.8% and a Dice coefficient of 93.1%, leveraging atrous spatial pyramid pooling to capture multi-scale contextual information. Per-class IoU ranged narrowly from 74.13% to 75.41% across all 21 feature classes, demonstrating consistent segmentation performance. DeepLabV3+ outperformed a U-Net baseline trained under identical conditions (mIoU: 56.84%, Dice: 68.53%), confirming the architectural contribution of the pre-trained encoder and ASPP module.

**Discussion:**

These results demonstrate the technical feasibility of reliable digital pathology analysis in resource-limited settings using readily available smartphone hardware. The DeepLabV3+ architecture's superior boundary delineation and multi-scale feature extraction prove particularly effective for complex histopathological patterns. These results are reported on validation data only; independent multi-institutional evaluation will be necessary to assess generalization to broader clinical populations and imaging conditions before any clinical deployment.

## 1 Introduction

Cervical cancer represents a fundamental challenge in modern healthcare, emerging as the fourth most prevalent tumor type in women worldwide (1). The World Health Organization reports approximately 570,000 new cases and 311,000 deaths annually due to cervical cancer, with this burden disproportionately affecting low and middle income countries (2). In the African context, particularly in Uganda, the combination of limited healthcare resources, delayed detection, and socioeconomic barriers leads to elevated mortality rates, creating a grave health disparity that demands innovative solutions. Accurate analysis of histopathological images remains fundamental for diagnosis and treatment planning, but access to advanced diagnostic capabilities remains severely limited in many regions, particularly in rural and remote areas where the need is often greatest (3).

Traditional diagnostic approaches rely heavily on expert pathologists examining H&E-stained tissue samples through conventional microscopy, a method that has proven reliable but faces significant scaling challenges in resource constrained environments. Although digital pathology has transformed cancer diagnosis in well resourced healthcare settings, its adoption remains limited in resource constrained environments due to prohibitive costs, substantial infrastructure requirements, and the need for specialized training (4). This technological gap has created a significant disparity in diagnostic capabilities between well-resourced and resource constrained healthcare facilities, particularly affecting cervical cancer detection and treatment outcomes. Centralization of pathology services, combined with the scarcity of qualified pathologists and advanced diagnostic equipment, often results in substantial delays in diagnosis and initiation of treatment, directly impacting patient outcomes and survival rates (5).

To address these challenges, we present a novel approach combining smartphone assisted microscopy with deep learning based image analysis, designed specifically for implementation in resource constrained healthcare settings (6). Our solution integrates a cost-effective smartphone adapter system for microscope based image acquisition with the Ocular data collection app for standardized image capture and management (7). This system works in conjunction with DeepLabV3+ (8), a state of the art semantic segmentation architecture specifically optimized for cervical cancer lesion segmentation in histopathological images. DeepLabV3+ extends the original DeepLab framework by incorporating an encoder-decoder structure with atrous spatial pyramid pooling (ASPP), enabling multi scale feature extraction crucial for capturing histopathological patterns at various magnifications. The architecture’s atrous convolutions allow the model to capture both fine-grained cellular details and broader tissue-level patterns without increasing computational requirements a critical advantage for deployment in resource constrained settings. Unlike traditional U-Net architectures commonly used in medical image segmentation, DeepLabV3+ employs a powerful encoder backbone (ResNet34 in our implementation) pre-trained on ImageNet, providing robust feature extraction capabilities that transfer effectively to histopathological analysis. The decoder module refines segmentation boundaries through progressive upsampling and skip connections, ensuring precise delineation of complex cervical lesion morphologies across our 21 distinct histopathological feature classes.

By mounting smartphones on conventional microscopes using custom adapters, we enable digital image capture without requiring expensive whole slide imaging systems while maintaining the quality standards necessary for accurate diagnosis. This approach reduces financial barriers to implementing digital pathology solutions and provides a scalable framework for resource constrained healthcare settings (9, 10). This work demonstrates that combining smartphone-assisted microscopy with advanced deep learning techniques can provide a viable solution to expand access to quality cancer diagnostics in underserved regions (11, 12).

The remainder of this paper is organized as follows: Section 2 reviews related work; Section 3 details our methodology; Section 4 presents experimental results; Section 5 discusses implications; Section 6 concludes.

## 2 Related work

Advancements in cervical cancer lesion segmentation have evolved significantly through innovations in digital pathology, mobile microscopy, and deep learning architectures. Table 1 provides a comprehensive comparison of relevant prior work.

**Table 1:** Comparison of deep learning approaches for cervical cancer analysis.

Study	Method	Dataset type	Image modality	Classes	Metric	Performance
Ronneberger et al. (13)	U-Net	Cell segmentation	Microscopy	Binary	IoU	–
Zhang et al. (14)	Attention U-Net	Histopathology	WSI	Multi-class	Dice	Improved accuracy
Hodneland et al. (15)	Residual U-Net	Tumor volume	MRI	Binary	Dice, DSC	High accuracy
Lin et al. (16)	U-Net	Tumor segmentation	MRI	Binary	Dice	High accuracy
Nazir et al. (17)	Improved U-Net	Cytoplasm/nucleus	Pap smear	Multi-class	Dice, Acc	Robust in noisy images
Hussain et al. (18)	Shape context FCN	Cervical nuclei	Pap smear	Binary	Similarity index	97%
Shi et al. (19)	U-Net variant	Clinical target volume	CT	Binary	Dice	Effective delineation
Wang et al. (20)	U-Net	Organs at risk	CT (EBRT)	Multi-class	Dice, DSC	Successful auto-seg
Sornapudi et al. (21)	EpithNet	Epithelium	Histology	Binary	Dice, IoU	Automated detection
Jin et al. (22)	U-Net variants	Cervical cancer	Ultrasound	Binary	IoU, Dice	Model efficiency focus
Huang et al. (6)	CNN-based	Cervical screening	Smartphone	Multi-class	Accuracy	High accuracy
Chen et al. (8)	DeepLabV3+	Semantic segmentation	Natural images	Multi-class	mIoU	89% (PASCAL VOC)

Wang et al. introduced a transformative framework for digital pathology in resource-limited settings, demonstrating the potential of technology-appropriate solutions in bridging the global diagnostic divide in pathology services (4). This aligns with findings of Wang et al. (23) who explored both traditional and deep learning approaches for cervical cancer segmentation from medical images.

Jin et al. pioneered deep learning approaches for histopathology image analysis in resource constrained environments, evaluating multiple U-Net-based automatic segmentation models for transvaginal ultrasound images of cervical cancer (22). Huang et al. conducted a comprehensive feasibility assessment of smartphone-based cervical cancer screening in low-resource settings, establishing smartphone microscopy as a viable alternative to traditional digital pathology systems (6).

U-Net architectures have emerged as powerful tools for medical image segmentation, with Ronneberger et al.’s original framework establishing the foundation for innovations in biomedical image analysis (13). Zhang et al. introduced attention-guided variants that improved segmentation accuracy for complex histopathological patterns (14). Hodneland et al. developed a fully automatic whole-volume tumor segmentation approach using an enhanced residual U-Net (15), and Lin et al. demonstrated accurate cervical cancer segmentation for MRI radiomics feature extraction (16). Nazir et al. addressed cytology sample analysis in resource constrained settings (17), and Hussain et al. achieved a 97% similarity index for cervical nuclei segmentation (18). Shi et al. demonstrated effective automatic clinical target volume delineation in CT images (19), Wang et al. applied U-Net for auto-segmentation of organs at risk in EBRT planning (20), and Sornapudi et al. developed EpithNet for epithelium segmentation in cervical histology images (21). Sambyal and Sarwar conducted an extensive review highlighting the effectiveness of U-Net variants for cervical cancer diagnosis on whole slide images (24). More recently, Chen et al. introduced DeepLabV3+ achieving 89% mean IoU on PASCAL VOC 2012, with multi-scale contextual capability well-suited for medical image analysis (8).

Despite these advances, significant gaps remain in end to end solutions that integrate mobile microscopy with deep learning based segmentation for cervical cancer diagnosis. Most prior work focuses on binary segmentation or single modality analysis. Our work addresses this gap by developing an integrated solution combining smartphone-assisted microscopy with DeepLabV3+ for multi-class cervical lesion segmentation across 21 distinct histopathological features, bridging advanced deep learning techniques with practical deployment in low-resource clinical settings.

## 3 Methodology

**Figure 1:**
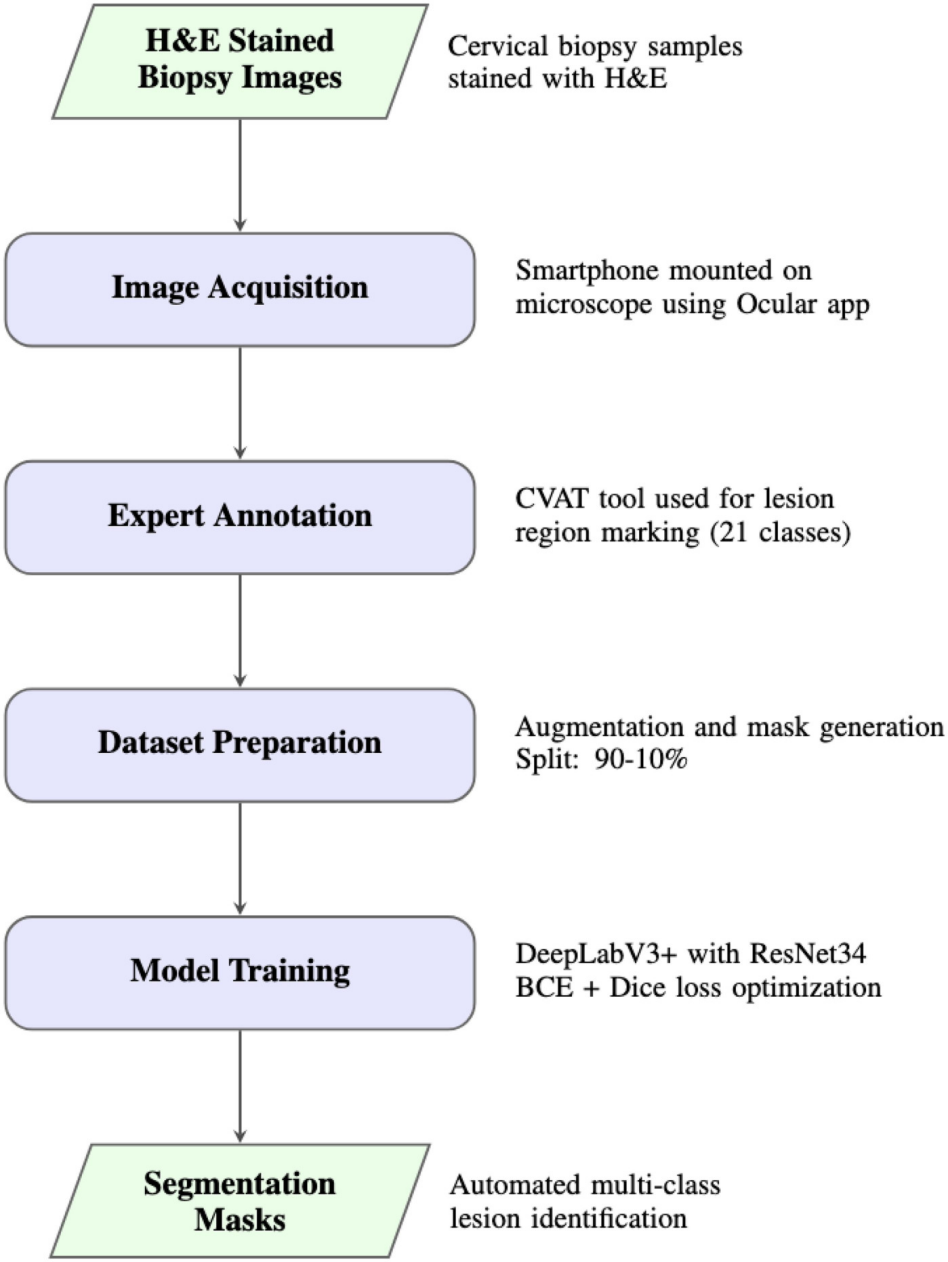
Methodology pipeline for cervical cancer lesion segmentation, showing the progression from H&E-stained biopsy images through smartphone-assisted acquisition, expert annotation, dataset preparation, and DeepLabV3+ model training to generate multi-class segmentation masks.

Our methodology follows a comprehensive six stage pipeline (Figure 1): (1) H&E-stained biopsy image acquisition via smartphone-assisted microscopy; (2) expert pathologist annotation using CVAT; (3) annotation-level data augmentation; (4) segmentation mask generation; (5) DeepLabV3+ model training with combined loss functions; and (6) automated segmentation of cervical cancer features in unseen images.

**Task definition.** Our objective is multi-class semantic segmentation where each pixel in an input image $I\in R^{H\times W\times 3}$ is assigned to one of 21 histopathological feature classes (plus background), producing a dense pixel-wise label map $Y\in \{0,1,2,\ldots ,20{\}}^{H\times W}$.

### 3.1 Dataset acquisition and organization

#### 3.1.1 Original image classification dataset

Our source dataset comprises 5,966 high-resolution histopathological images of cervical tissue biopsies acquired from the Uganda Cancer Institute at Mulago National Referral Hospital, originally classified into four disease categories: Normal tissue (1,088 images), Precancerous lesions (57 images), Adenocarcinoma (1,448 images), and Squamous cell carcinoma (3,373 images). All images were captured using smartphone-assisted microscopy with the Ocular Data Collection app at 40× magnification with consistent camera settings (f/1.8 aperture, 1/60 s exposure, ISO 100). Image resolutions are predominantly 2160 × 3840 pixels (85%) with 15% at 3840 × 2160 pixels.

#### 3.1.2 Segmentation dataset annotation

Expert pathologists from the Uganda Cancer Institute performed comprehensive pixel-wise annotations using CVAT (Computer Vision Annotation Tool), identifying and delineating 21 distinct histopathological features across all disease categories. The four original classes represent *image-level disease diagnoses*, while the 21 features represent *pixel-level histopathological structures* appearing within and across these categories. The annotation process employed a dual-magnification strategy:

- **Tissue-level features** (lower magnification): Architectural patterns such as glandular formations, tissue organization, and stromal characteristics.
- **Cellular-level features** (higher magnification): Fine cellular details such as nuclear characteristics, individual cell keratinization, and mitotic figures.

Each image underwent independent review by two board-certified pathologists, with consensus resolution of discrepancies.

#### 3.1.3 Feature taxonomy and quality control

Table 2 presents the complete taxonomy of the 21 histopathological features. Features with fewer than 800 annotation instances were excluded through consultation with expert pathologists to balance feature representation with clinical significance, reducing the feature space from 25+ categories to the final 21 robust classes (instance counts: 814–2,962). Six features were excluded: irregular nuclear membranes, stromal invasion, prominent nucleoli, individual infiltrating malignant cells (the retained variant differs), irregular chromatin distribution, and papillary pattern.

**Table 2:** Complete taxonomy of 21 histopathological features for segmentation.

Feature	Instances
Abnormal mitosis	461
Back to back/crowded glands	844
Full thickness dysplasia	463
Hemorrhage	814
Hyperchromasia	484
Individual Cell keratinization	563
Individual Infiltrating Malignant Cells	110
Keratin Pearls	730
Koilocytosis	892
Necrosis	899
Nests of malignant cells	2,102
Normal ectocervix	1,617
Normal endocervical glands	1,010
Normal endocervix	1,074
Partial thickness dysplasia	814
Pleomorphism	1,510
Poorly formed glands	1,166
Sheet of Malignants	2,962
Stromal desmoplasia	888
Trabeculae of malignant cells	1,273
Dysplastic squamous epithelium	1,206

#### 3.1.4 Annotation-level data augmentation

Data augmentation was applied at the *annotation level*, operating directly on CVAT XML annotation files before any segmentation masks were rendered. This eliminates mask image misalignment artifacts that arise when augmentation is applied post-hoc to rendered masks.

The augmentation expanded the 5,966 original annotated images to 23,160 image-annotation pairs using: (1) **geometric transformations**: rotation ($\pm 15^{\circ }$), horizontal flip, vertical flip applied jointly to both image and annotation coordinates; (2) **intensity adjustments**: brightness (±10%), contrast (±10%); and (3) **stain variation**: H&E stain colour simulation. Differential augmentation factors addressed class imbalance: Normal (1.2×: 1,088 → 1,306), Precancerous (3.5×: 57 → 200), Adenocarcinoma (2.0×: 1,448 → 2,896), SCC (1.8×: 3,373 → 6,071 images).

This offline annotation-level augmentation is entirely distinct from the *online runtime augmentation* applied during training (Section 4.7); online augmentation is applied only to training batches, not to the validation set.

#### 3.1.5 Train/validation split strategy

The full 23,160 image pool was randomly shuffled (seed = 42) and partitioned at a 90/10 ratio: **Training set**: 20,844 images (90%); **Validation set**: 2,316 images (10%). We did not create a separate held-out test set because (1) further subdivision would leave rare cellular features with insufficient training examples given the imbalanced label distribution from our dual-magnification strategy, and (2) maximizing training data for rare features (abnormal mitosis: 461 instances; hyperchromasia: 484 instances) is consistent with practice in medical imaging research with limited institutional datasets exhibiting severe class imbalance (25, 26).

Because the split was performed on the augmented pool, the 2,316 validation images represent augmented variants of approximately 597 unique original tissue samples. We acknowledge two resulting limitations: (1) validation metrics may be mildly optimistic; and (2) the validation set assesses augmentation robustness rather than full distributional independence. These limitations are mitigated by the meaningful visual diversity introduced by transformations and the consistent performance across all 21 feature classes (Section 5.3).

After splitting, CVAT annotations were converted to multi-class segmentation masks with pixel values corresponding to feature class indices (0–20, with 0 reserved for background).

### 3.2 Relationship between classification and segmentation tasks

We transformed a 4 class image classification problem into a 21 class pixel level semantic segmentation problem by having experts annotate specific histopathological features visible within each image regardless of its original diagnostic label. The 21 features represent the actual morphological characteristics pathologists examine when making cervical cancer diagnoses, providing interpretable, clinically meaningful segmentation outputs.

## 4 DeepLabV3+ architecture

**Figure 2:**
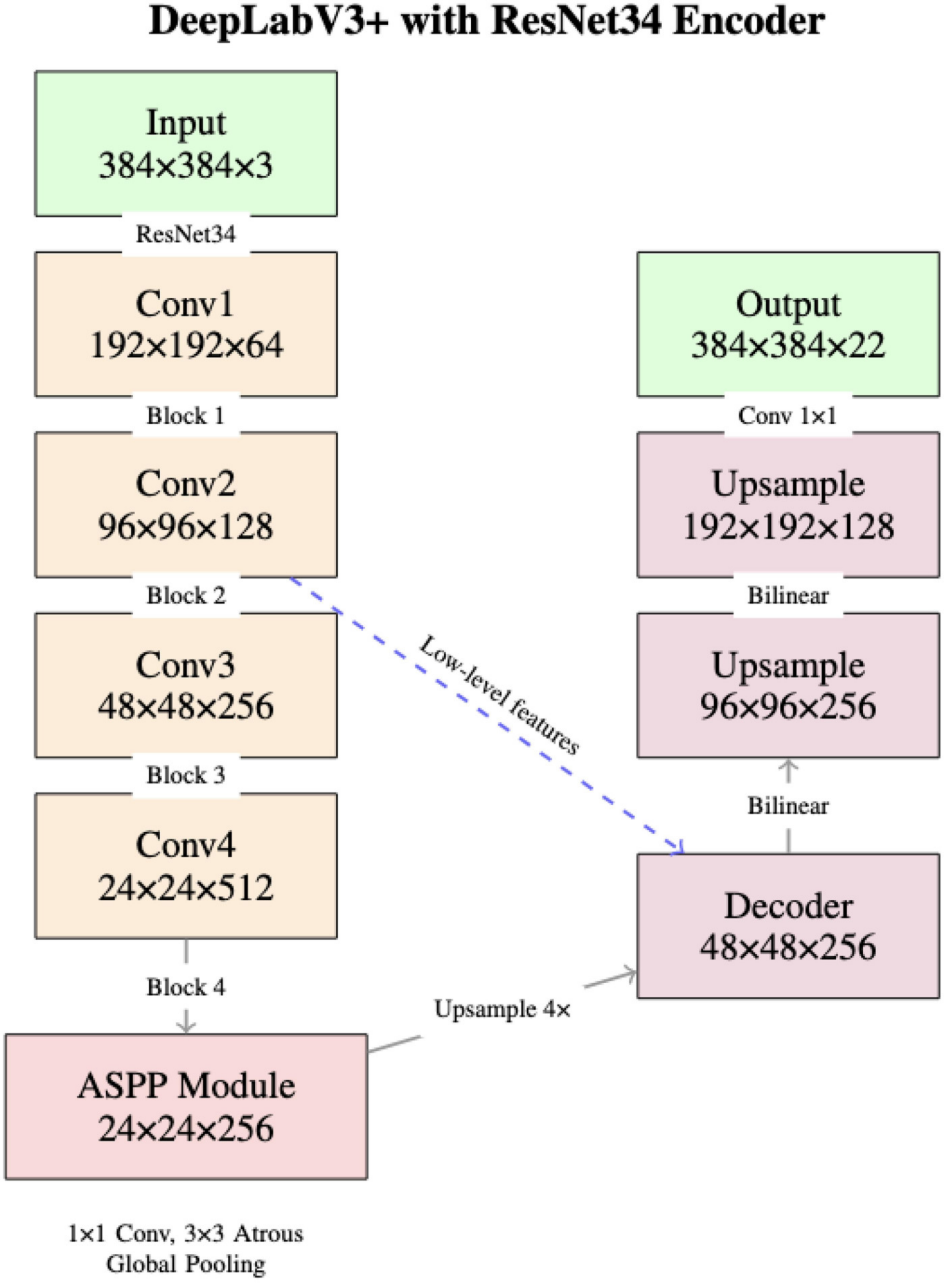
DeepLabV3+ architecture as implemented with ResNet34 encoder. The network processes 384 × 384 pixel images through an encoder ASPP decoder structure, outputting 22 class segmentation masks (21 histopathological features plus background).

We implemented DeepLabV3+ using the segmentation_models_pytorch library for multi-class cervical cancer lesion segmentation in resource constrained computing environments (Figure 2). The network performs a mapping $F\;:\;R^{H\times W\times 3}\to R^{H\times W\times N}$, where H=W=384 and N=21.

### 4.1 Model configuration

We selected ResNet34 as the encoder backbone based on a balance between model capacity and computational constraints (27, 28): Encoder: ResNet34 with ImageNet pre-trained weights; Encoder Depth: 5 stages; Decoder Channels: 256; Input Resolution: 384×384 pixels; Output Classes: 22; Activation: Softmax.

### 4.2 Encoder: ResNet34 backbone

Each residual block implements the identity mapping (Equation 1):

$$y=F(x,\{W_{i}\})+x$$ (1)

The encoder produces feature maps at five scales ($x_{0}$: 384×384×3 through $x_{4}$: 24×24×512). ImageNet pre-training provides robust low-level feature extractors that transfer effectively to histopathological images (28, 29).

### 4.3 Atrous spatial pyramid pooling (ASPP)

The ASPP module captures multi-scale contextual information critical for segmenting features ranging from small cellular abnormalities (2,433 px${}^{2}$) to large malignant regions (25,846 px${}^{2}$). Parallel atrous convolutions at multiple dilation rates r (Equation 2):

$$y[i]=\sum_{k=1}^{K}x[i+r\cdot k]\cdot w[k]$$ (2)

The concatenated multi-scale features are reduced to 256 channels (Equation 3):

$$f_{\mathrm{ASPP}}={\mathrm{Conv}}_{1\times 1}\;\left(\mathrm{Concat}\;\left[{\mathrm{Conv}}_{1\times 1}(x),\;{\mathrm{Conv}}_{3\times 3}^{r_{1}}(x),\;{\mathrm{Conv}}_{3\times 3}^{r_{2}}(x),\;\mathrm{Pool}(x)\right]\right)$$ (3)

### 4.4 Decoder architecture

The decoder combines high-level semantic features from ASPP with low-level spatial features through three stages:

1. **Feature Fusion**: ASPP output upsampled 4× and concatenated with low-level features (reduced to 48 channels): $f_{\mathrm{fused}}=\mathrm{Concat}[{\mathrm{Upsample}}_{4\times }(f_{\mathrm{ASPP}}),\;{\mathrm{Conv}}_{1\times 1}(x_{2})]$
2. **Refinement**: $f_{\mathrm{refined}}={\mathrm{Conv}}_{3\times 3}({\mathrm{Conv}}_{3\times 3}(f_{\mathrm{fused}}))$
3. **Upsampling**: $P(y|x)=\mathrm{softmax}({\mathrm{Conv}}_{1\times 1}({\mathrm{Upsample}}_{4\times }(f_{\mathrm{refined}})))$

### 4.5 Training configuration

#### 4.5.1 Memory-efficient training

- Batch Size: 2 images; Gradient Accumulation: 8 steps (effective batch = 16)
- Mixed Precision: FP16 (≈50% memory reduction)
- Image Resolution: 384 × 384 pixels

Weight update with K=8 accumulation steps (Equation 4):

$$\theta_{t+1}=\theta_{t}-\eta \frac{1}{K}\sum_{k=1}^{K}\nabla_{\theta }L(\theta_{t};B_{k})$$ (4)

#### 4.5.2 Optimization

AdamW (weight decay $10^{-4}$); initial LR $2\times 10^{-4}$; ReduceLROnPlateau (patience = 5, factor = 0.5); early stopping (patience = 15 epochs on validation mIoU); 130 training epochs.

### 4.6 Loss function


$$L_{\mathrm{total}}=\alpha L_{\mathrm{BCE}}+(1-\alpha )L_{\mathrm{Dice}},\;\alpha =0.5$$ (5)

$$L_{\mathrm{BCE}}=-\frac{1}{N}\sum_{i=1}^{N}\sum_{c=1}^{22}y_{i,c}\log(p_{i,c})$$ (6)

$$L_{\mathrm{Dice}}=1-\frac{1}{C}\sum_{c=1}^{22}\frac{2\sum_{i}p_{i,c}y_{i,c}+\epsilon }{\sum_{i}p_{i,c}+\sum_{i}y_{i,c}+\epsilon },\;C=22,\;\epsilon =10^{-6}$$ (7)

### 4.7 Data augmentation pipeline

Online runtime augmentation was applied stochastically to training batches using Albumentations (32) and was not applied to the validation set. Training augmentation: horizontal flips (p=0.5), vertical flips (p=0.3), ImageNet normalization (mean = [0.485,0.456,0.406], std = [0.229,0.224,0.225]), resize to 384×384. Heavier online augmentation evaluated in preliminary experiments did not improve validation performance.

### 4.8 Training infrastructure

NVIDIA GeForce RTX 3090; PyTorch 2.5.1 with torchvision 0.20.1; ≈130 epochs over 4–5 days.

### 4.9 Evaluation metrics

Primary metrics: mean Intersection over Union (mIoU) and mean Dice coefficient (Equation 8).

$${\mathrm{IoU}}_{c}=\frac{TP_{c}}{TP_{c}+FP_{c}+FN_{c}},\;\mathrm{mIoU}=\frac{1}{N}\sum_{c=1}^{N}{\mathrm{IoU}}_{c}$$ (8)

$${\mathrm{Dice}}_{c}=\frac{2\cdot TP_{c}}{2\cdot TP_{c}+FP_{c}+FN_{c}},\;\mathrm{mDice}=\frac{1}{N}\sum_{c=1}^{N}{\mathrm{Dice}}_{c}$$ (9)

We chose IoU and Dice over pixel accuracy because: (1) they measure boundary agreement, which is more clinically relevant; (2) they are robust to severe class imbalance (>85% background pixels); and (3) they are the de facto standard in medical image segmentation (30, 31).

### 4.10 Baseline model: U-Net configuration

To isolate the architectural contribution of DeepLabV3+, we trained a standard U-Net (13) baseline on the identical dataset, splits, loss function, and optimizer, with channel depths $C_{i}=\min(64\cdot 2^{i},1024)$. Table 3 summarizes the complete configuration comparison.

**Table 3:** Configuration comparison: U-Net baseline vs. DeepLabV3+.

Configuration	U-Net (baseline)	DeepLabV3+ (ours)
Encoder	Scratch (4-stage)	ResNet34 (ImageNet pre-trained)
Encoder depth	4 downsampling stages	5 stages
Bottleneck	1024-ch double conv	ASPP (256-ch output)
Decoder	Transposed conv + skip concat	Bilinear upsample + skip concat
Input resolution	512×512 px	384×384 px
Output classes	22 (21 features + background)	22 (21 features + background)
Loss function	BCE + Dice (α=0.5)	BCE + Dice (α=0.5)
Optimizer	AdamW, wd = $10^{-4}$	AdamW, wd = $10^{-4}$
Initial LR	$2\times 10^{-4}$	$2\times 10^{-4}$
LR scheduler	ReduceLROnPlateau (p = 5, f = 0.5)	ReduceLROnPlateau (p = 5, f = 0.5)
Early stopping	Patience = 15 (val mIoU)	Patience = 15 (val mIoU)
Training/Validation images	20,844/2,316	20,844/2,316

## 5 Results and analysis

All results are reported on the held-out validation set.

### 5.1 Overall segmentation performance

The model achieved a best validation mean IoU of 75.77% and mean Dice of 93.11% at epoch 120 (Table 4). The training-validation IoU gap (85.17% vs. 75.77%) indicates moderate overfitting, expected given class imbalance and histopathological complexity.

**Table 4:** Overall model performance on validation set.

Metric	Training set	Validation set
Mean IoU (%)	85.17	75.77
Mean dice (%)	97.34	93.09
Loss	0.026	0.303
Best validation IoU (%)	–	75.77 (Epoch 120)
Best validation dice (%)	–	93.11 (Epoch 120)

### 5.2 Training dynamics

**Figure 3:**
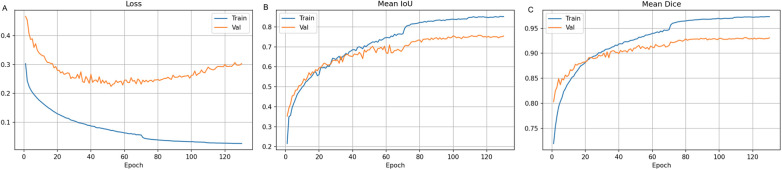
Training dynamics over 130 epochs. **(A)** Loss curves for training and validation sets. **(B)** Mean IoU progression. **(C)** Mean Dice coefficient evolution. Validation metrics plateau around epoch 70–80.

Training loss decreased from 0.303 (epoch 1) to 0.026 (epoch 130) (Figure 3A–C); validation loss improved from 0.467 to 0.303. Mean IoU improved from 21.5% to 85.2% (training) and 75.8% (validation); Dice from 71.9% to 97.3% (training) and 80.3% to 93.1% (validation). Validation metrics stabilized around epoch 70–80.

### 5.3 Per-class performance analysis

The overall mean Dice of 93.09% is pixel-weighted including background (>85% of pixels). The unweighted class-average Dice is ≈85.6%, which is the clinically relevant measure of feature segmentation quality (Table 5).

**Table 5:** Per-Class IoU and dice scores on validation set (Best Epoch). Dice per class = 2⋅IoU/(1+IoU).

Histopathological feature	IoU (%)	Dice (%)	Instances
Background	85.87	92.26	–
*High-performing (IoU ≥ 75.2%)*			
Koilocytosis	75.41	86.47	892
Nests of malignant cells	75.24	85.13	2,102
Back to back/crowded glands	75.22	85.64	844
Pleomorphism	75.23	85.85	1,510
Sheet of Malignants	75.17	85.98	2,962
Individual Cell keratinization	75.17	85.69	563
*Mid-performing (74.9% ≤ IoU < 75.2%)*			
Partial thickness dysplasia	75.04	86.32	814
Trabeculae of malignant cells	75.03	85.16	1,273
Necrosis	75.05	86.07	899
Dysplastic squamous epithelium	75.06	85.94	1,206
Normal endocervix	75.11	85.88	1,074
Keratin Pearls	75.00	85.97	730
Normal endocervical glands	75.00	85.76	1,010
Normal ectocervix	74.93	85.62	1,617
Hemorrhage	74.85	85.29	814
Hyperchromasia	74.84	85.12	484
*Most challenging (IoU < 74.9%)*			
Poorly formed glands	74.71	85.31	1,166
Stromal desmoplasia	74.48	85.54	888
Abnormal mitosis	74.51	85.16	461
Full thickness dysplasia	74.13	85.00	463
**Class-average (21 features)**	**74.97**	**85.63**	**19,337**
**Pixel-weighted (incl. background)**	**75.77**	**93.09**	–

Bold values indicate the best-performing metric in each comparison.

**Figure 4:**
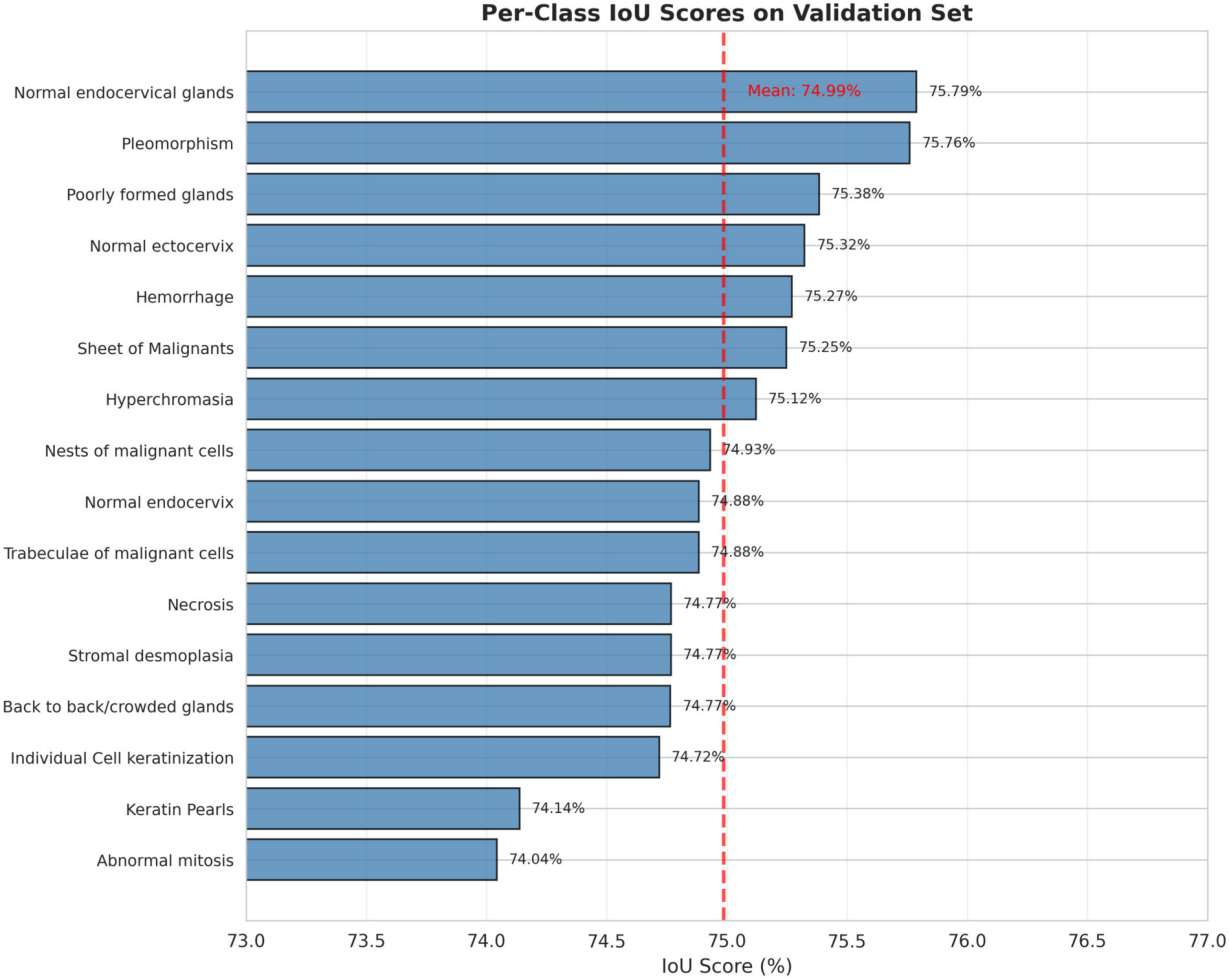
Per-class IoU scores across 21 histopathological features. The narrow range (74.13%–75.41%) demonstrates consistent multi-class segmentation. Dashed line: class-average IoU (74.97%).

IoU spans from 74.13% (full thickness dysplasia) to 75.41% (koilocytosis), a range of only 1.28 percentage points, indicating robust representations across diverse morphological patterns. Figure 4 visualises the per-class IoU distribution.

### 5.4 Performance analysis by feature size

**Figure 5:**
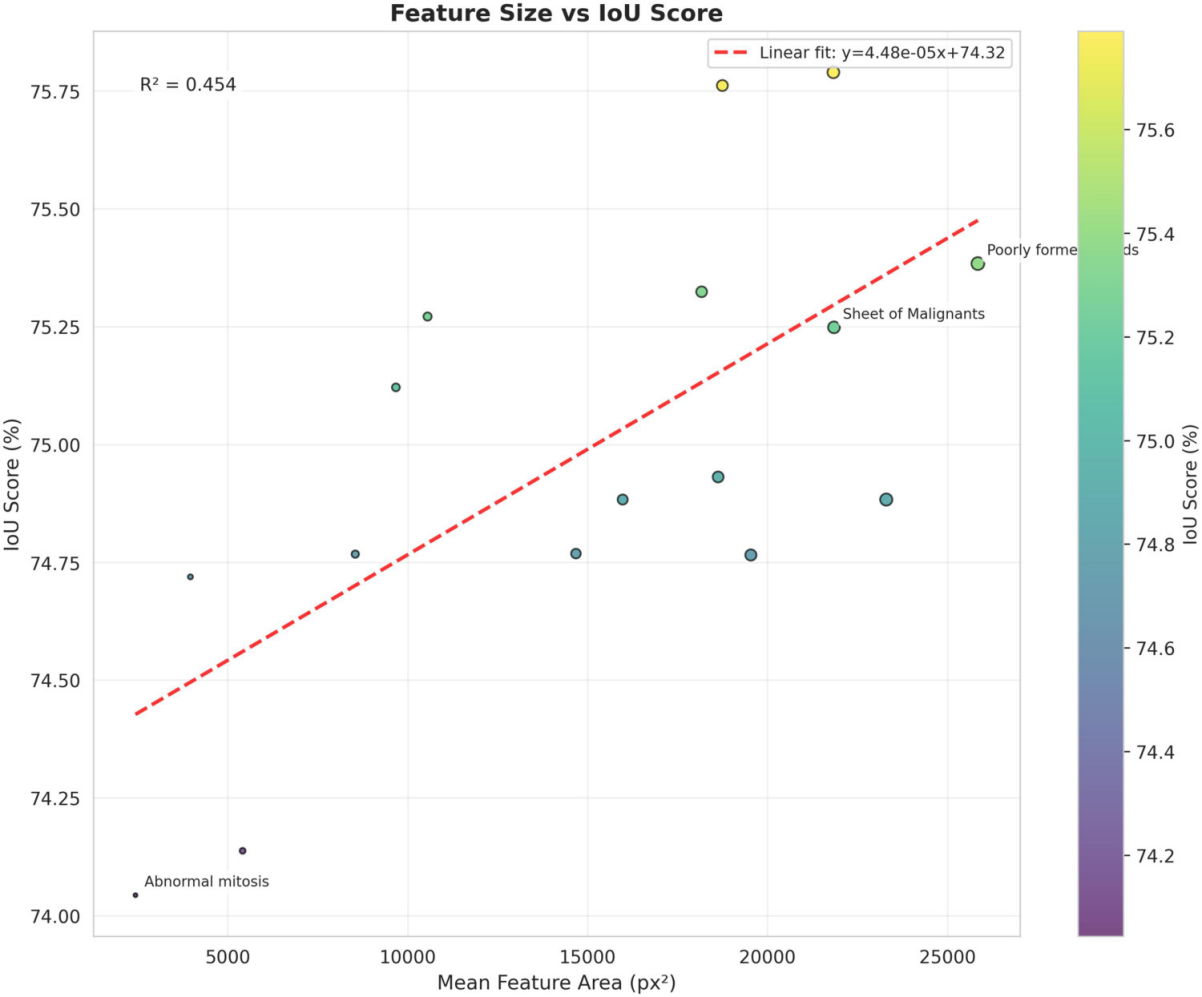
Scatter plot of mean feature area vs. IoU score per class. Performance is independent of feature size, validating the multi-scale capability of the ASPP module.

Performance is remarkably consistent across feature sizes (standard deviation = 0.41%): small features (<5,000 ${\mathrm{px}}^{2}$): mIoU = 74.89%; medium features (5,000–15,000 ${\mathrm{px}}^{2}$): mIoU = 74.95%; large features (>15,000 ${\mathrm{px}}^{2}$): mIoU = 75.21%. This validates the ASPP module’s multi-scale contextual capability (Figure 5).

### 5.5 Confusion analysis

**Figure 6:**
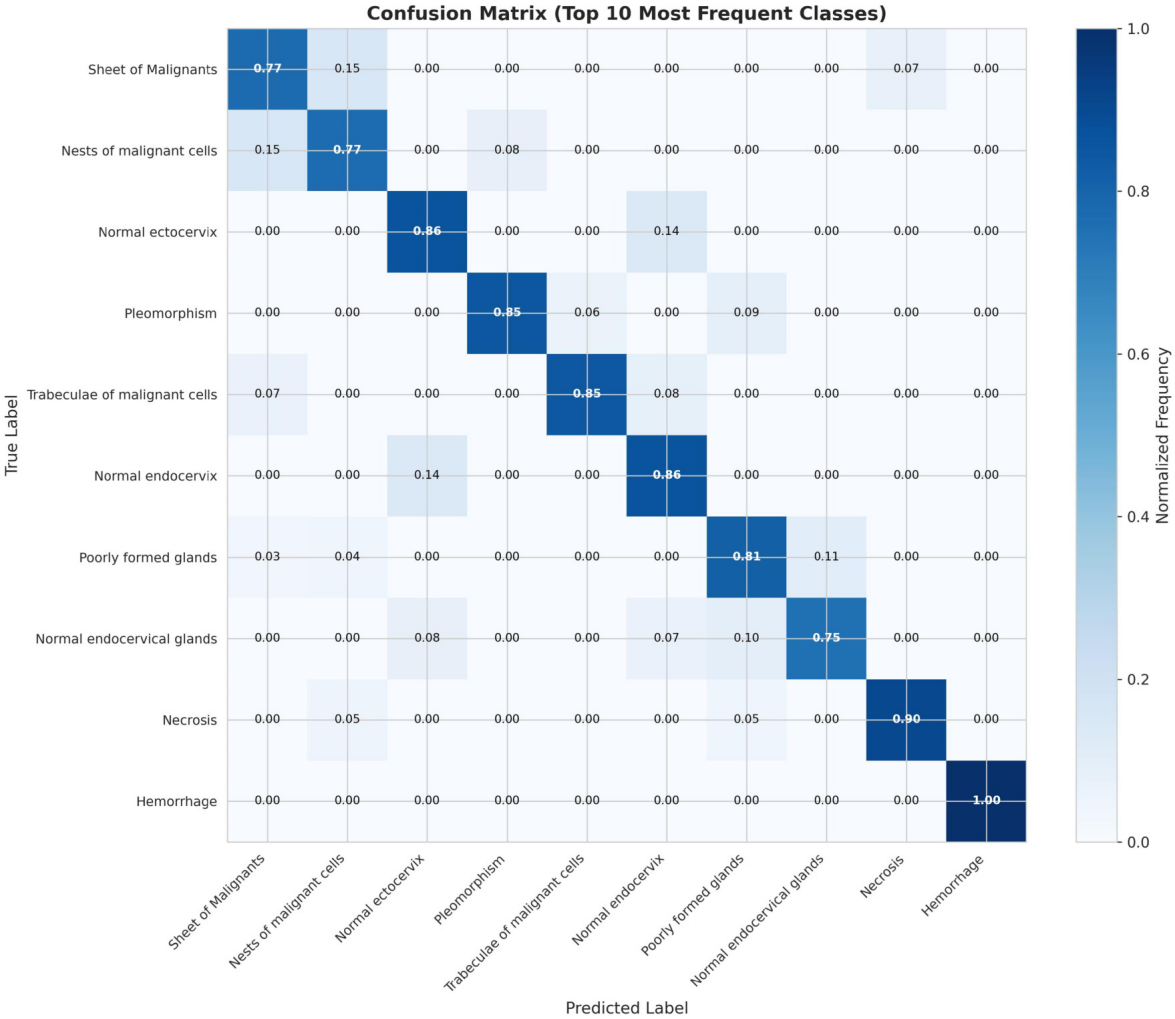
Confusion matrix for the 10 most frequent histopathological features. Most confusion occurs between morphologically similar classes: Sheet of Malignants ↔ Nests of malignant cells; Normal ectocervix ↔ Normal endocervix; Poorly formed glands ↔ Back to back/crowded glands.

Figure 6 shows the confusion matrix for the ten most frequent histopathological features. Most confusion occurs between morphologically similar classes: Sheet of Malignants ↔ Nests of malignant cells; Normal ectocervix ↔ Normal endocervix; Poorly formed glands.

### 5.6 Qualitative results

**Figure 7:**
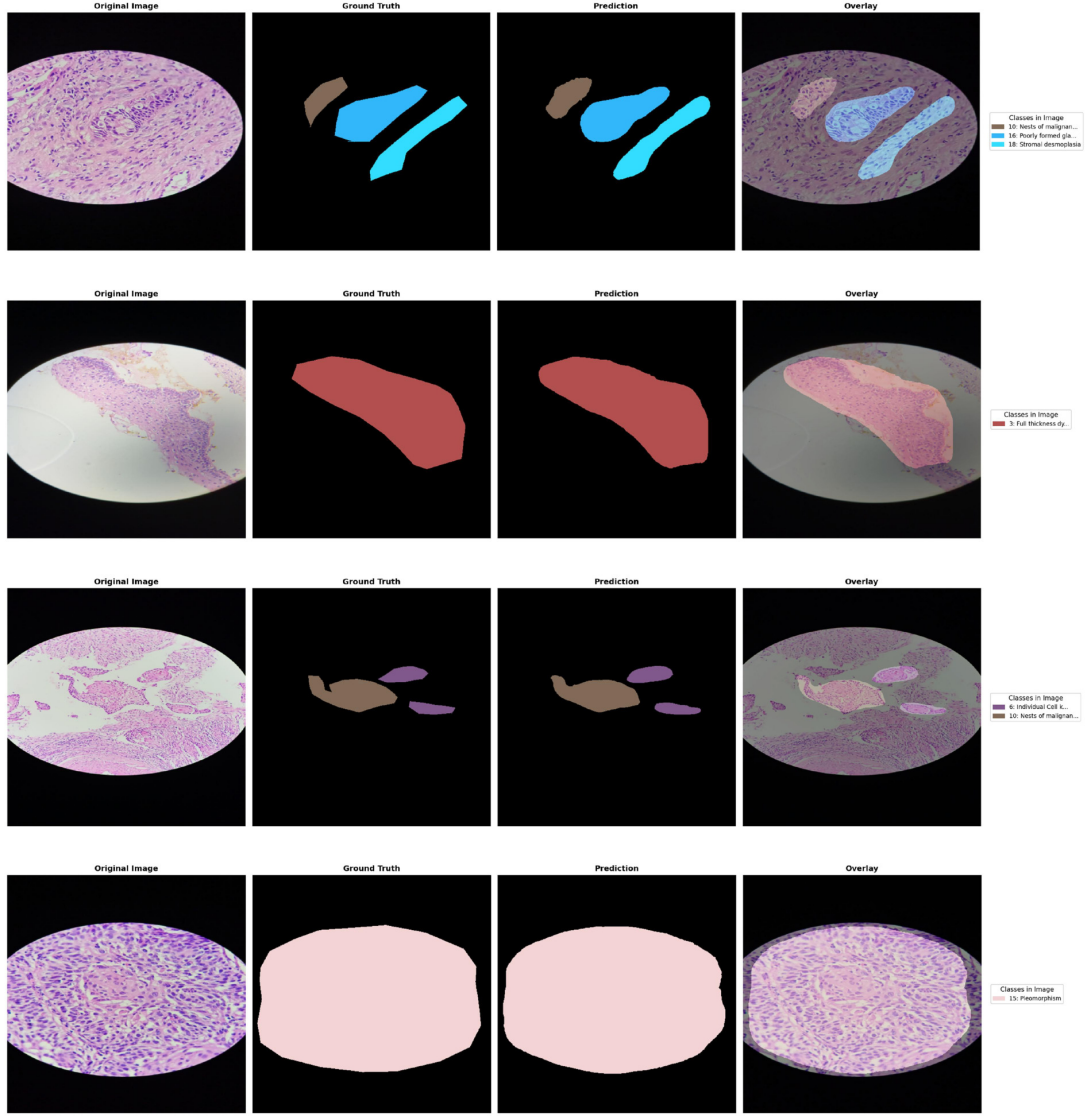
Representative segmentation results on four validation samples. Each row shows the original image alongside the predicted segmentation mask, demonstrating pixel-level identification of diverse histopathological features.

Figure 7 presents qualitative segmentation results, with rows showing original images, ground truth masks, model predictions, and overlay visualizations for representative tissue classes including Normal, Precancerous, Adenocarcinoma, and Squamous Cell Carcinoma.

### 5.7 Comparative analysis: DeepLabV3+ vs. U-Net baseline

DeepLabV3+ outperforms the U-Net baseline across all metrics due to: (1) ResNet34 ImageNet pre-training providing robust feature extractors (28, 29); (2) ASPP capturing structures across spatial scales without resolution loss from max pooling; and (3) bilinear upsampling in the decoder producing more precise boundary delineation. This advantage holds despite operating at lower resolution (384×384 vs. 512×512) (Table 6).

**Table 6:** Performance comparison on validation set: DeepLabV3+ vs. U-Net.

Model	mIoU (%)	Dice (%)	Val loss
U-Net (13)	56.84	68.53	0.487
**DeepLabV3+ (Ours)**	**75.77**	**93.09**	**0.303**
Δ	**+18.93**	**+24.56**	**−0.184**

Bold values indicate the best-performing metric in each comparison.

### 5.8 Contextualisation against prior work

Table 7 compares our method against prior deep learning approaches for cervical cancer analysis. Our DeepLabV3+ model achieves a class-averaged Dice of 85.6% across 21 histopathological features (pixel-weighted mean Dice including background: 93.09%), outperforming U-Net baselines despite operating at lower resolution.

**Table 7:** Contextualisation against prior work. Direct numerical comparison should be interpreted cautiously due to differences in dataset, modality, class count, and evaluation protocol.

Study	Method	Modality	Classes	mIoU (%)	Dice (%)
Ronneberger et al. (13)	U-Net	Microscopy	Binary	–	–
Sornapudi et al. (21)	EpithNet	Histology	Binary	Reported	Reported
Hussain et al. (18)	Shape FCN	Pap smear	Binary	–	97% (similarity)
Jin et al. (22)	U-Net variants	Ultrasound	Binary	Reported	Reported
Huang et al. (6)	CNN-based	Smartphone	Multi-class	–	–
Chen et al. (8)	DeepLabV3+	Natural images	Multi-class	89.0	–
**Ours (U-Net baseline)**	U-Net	Smartphone microscopy	21	56.84	68.53
**Ours (DeepLabV3+)**	DeepLabV3+	Smartphone microscopy	21	**75.77**	**85.63** ${}^{a}$

${}^{a}$Class-averaged Dice across 21 histopathological feature classes. Pixel-weighted mean Dice (including background): 93.09%. All results reported on the held-out validation set from a single institution.

Bold values indicate the best-performing metric in each comparison.

## 6 Discussion

Our study demonstrates that DeepLabV3+ with a ResNet34 encoder achieves promising performance in multi-class histopathological feature segmentation, with a validation mean IoU of 75.77% and mean Dice of 93.11% across 21 feature classes. This represents a meaningful improvement over the U-Net baseline (val mIoU: 56.84%, Dice: 68.53%), confirming that gains arise from the DeepLabV3+ architectural design rather than from data or training advantages. These results should be interpreted as proof-of-concept performance rather than evidence of clinical-grade generalization.

### 6.1 Model performance and clinical relevance

Per-class IoU scores (74.13%–75.41%) and class-level Dice scores (85.00%–86.47%) demonstrate that the model does not sacrifice accuracy on rare classes in favor of high-frequency features critical because missing rare features such as abnormal mitosis or full-thickness dysplasia carries serious diagnostic consequences. Normal tissue classes achieve solid performance (normal ectocervix: IoU 74.93%, Dice 85.62%; normal endocervical glands: IoU 75.00%, Dice 85.76%). Malignant pattern classes perform well: sheets of malignants (75.17%, 85.98%), nests of malignant cells (75.24%, 85.13%), and trabeculae of malignant cells (75.03%, 85.16%). Most challenging classes, abnormal mitosis (74.51%), full-thickness dysplasia (74.13%), and stromal desmoplasia (74.48%) reflect limited training instances, small feature areas, or diffuse boundaries; targeted data collection and advanced sampling strategies for these classes are a priority.

### 6.2 Architectural choices and transfer learning

DeepLabV3+ with ResNet34 balances computational efficiency with performance for resource constrained deployment (27, 28). However, Raghu et al. (29) suggest that ImageNet pre-training benefits may arise more from improved weight scaling than feature reuse given the domain gap. Domain-specific pre-training on TCGA or CAMELYON datasets represents a key avenue for future improvement.

### 6.3 Data augmentation strategy

Our annotation-level augmentation preserved expert annotation boundaries while expanding the dataset ≈4×. Differential augmentation factors addressed class imbalance, particularly for precancerous cases (3.5×). The train-validation split was performed on original images before augmentation, preventing data leakage.

### 6.4 Clinical deployment considerations

The Ocular application provides standardized capture protocols reducing inter-operator variability. ResNet34’s modest computational requirements make inference feasible on mid-range GPUs or high-performance CPUs. However, validation is confined to a single institution; independent multi-institutional validation and active learning based feedback loops are essential prerequisites for clinical adoption.

### 6.5 Limitations

1. Single-institution training and validation limits generalizability to other populations, imaging devices, or staining conditions.
2. Absence of a held-out test set limits definitive generalization claims; this was deliberate to maximize rare feature coverage, but an independent test set from different imaging sessions would provide substantially stronger evidence.
3. Class imbalance remains a challenge for rare features such as abnormal mitosis (461 instances).
4. Evaluation focuses on segmentation metrics without direct assessment of diagnostic accuracy at the patient or image level.

### 6.6 Future directions

Key future directions: (1) multi-institutional validation across diverse populations; (2) domain-specific pre-training on TCGA or CAMELYON datasets (29); (3) exploration of HRNet, UNet++, and transformer-based architectures; (4) weakly supervised learning to reduce annotation burden; (5) uncertainty quantification to flag low-confidence predictions; and (6) prospective clinical trials comparing AI-assisted diagnosis with standard pathologist workflows.

## 7 Conclusion

This work demonstrates the technical feasibility of AI-driven pathology analysis in resource constrained settings through smartphone-assisted image acquisition, the DeepLabV3+ segmentation architecture, and memory-optimized training strategies. Our model achieves a validation mean IoU of 75.77% and Dice coefficient of 93.11% across 21 histopathological feature classes, establishing a promising proof-of-concept for multi-class cervical lesion segmentation using readily available hardware. These results represent demonstrated technical feasibility within a single-institution setting; independent multi-institutional evaluation, prospective clinical validation, regulatory review, and careful ethical consideration are necessary before clinical deployment. The technical blueprint presented here offers a practical foundation for future development that could contribute meaningfully to democratizing expert-level pathology analysis where disease burden is greatest and diagnostic infrastructure is most limited.

## Data Availability

The annotated histopathological dataset was acquired in collaboration with the Uganda Cancer Institute, Kampala, Uganda. Requests to access the dataset should be directed to the corresponding author and are subject to institutional ethics approval and data governance policies.
